# Dual-sided closure of an iatrogenic gastrocolonic fistula with over-the-scope clips

**DOI:** 10.1055/a-2351-2909

**Published:** 2024-07-15

**Authors:** Sunil Gupta, Roxana Chis, Jeffrey D. Mosko, Christopher Teshima

**Affiliations:** 1 10071 Therapeutic Endoscopy, St. Michaelʼs Hospital, Toronto, Canada; 2 8539 Gastroenterology and Hepatology, Westmead Hospital, Westmead, Australia

An 86-year-old man with walled-off pancreatic necrosis (WON) was initially managed with a percutaneous drain. He was referred to our service because of a persistent collection. After fluid had been instilled via the drain to expand the collapsed collection, endoscopic ultrasound (EUS)-guided cystogastrostomy was performed with a lumen-apposing metal stent (LAMS; 15-mm wide × 10 mm in length). A computed tomography (CT) scan the following week revealed a gastrocolostomy due to stent misdeployment. As the patient was clinically stable, we opted to wait 4 weeks to facilitate tract maturation prior to LAMS removal and definitive closure 1 .

During gastroscopy, the LAMS was visualized in the gastric body ( Video 1 ). The stent was easily traversed and the colon was entered, confirming the presence of the gastrocolostomy. To serve as an endoscopic marker, a through-the-scope (TTS) clip was placed on the colonic wall contralateral to the LAMS ( Fig. 1 ). The LAMS was then removed with a grasping forceps and the ensuing gastrocolonic fistula was visualized ( Fig. 2 **a** ). An over-the-scope (OTS) clip was successfully deployed to close the gastric side of the fistula ( Fig. 2 **b** ), with fluoroscopy confirming the absence of a contrast leak. A colonoscopy was then performed, with the previously placed TTS clip visualized in the transverse colon. After the corresponding colonic segment had been cleaned, a large fistula opening was noted ( Fig. 2 **c** ); although contrast did not traverse into the stomach, it deeply filled the fistulous tract. Given its size and depth, we opted to definitively close the colonic side of the fistula with a second OTS clip ( Fig. 2 **d** ), with contrast injection confirming the absence of contrast flow into the tract, indicating successful dual-sided closure ( Fig. 3 ).

**Fig. 1:**
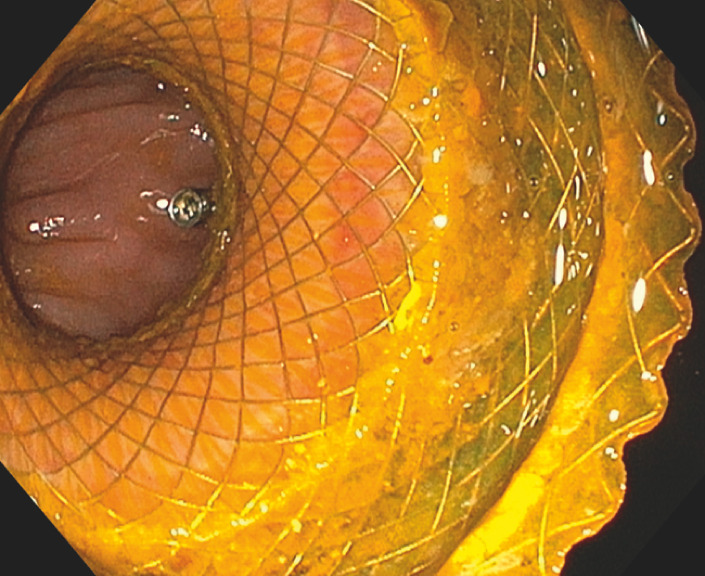
View during gastroscopy showing the gastrocolostomy stent with a through-the-scope clip placed on the colonic mucosa to serve as an endoscopic marker.

**Fig. 2:**
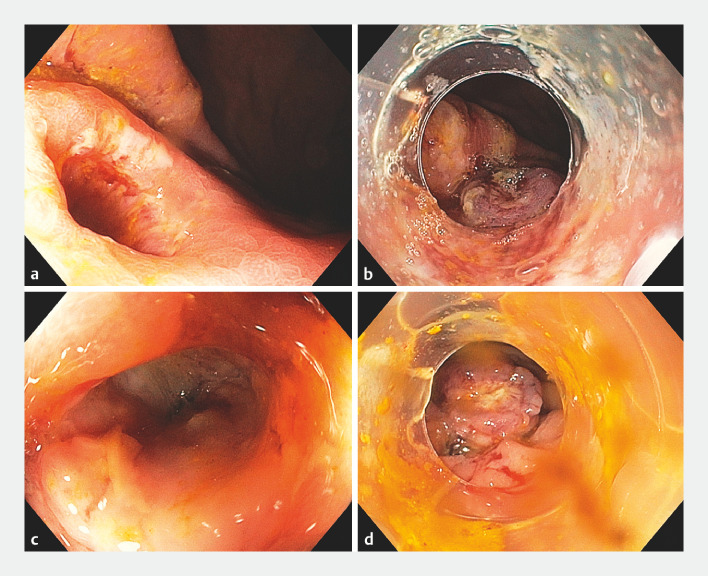
Images during gastrocolonic fistula closure showing the appearance from: **a, b** the gastric side of the fistula; **c, d** the colonic side of the fistula; **a, c** before closure; **b, d** after closure with over-the-scope clips.

**Fig. 3:**
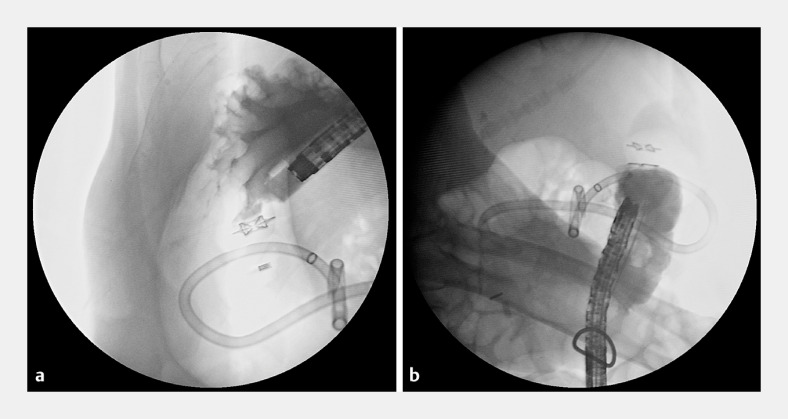
Fluoroscopic images demonstrating the absence of a contrast leak, indicating successful dual-sided closure of the gastrocolonic fistula.

Dual-sided closure of an iatrogenic gastrocolonic fistula with over-the-scope clips after lumen-apposing metal stent misdeployment during drainage of walled-off pancreatic necrosis.Video 1

A subsequent sinogram via a percutaneous drain revealed a WON-to-colon fistula. This may have contributed to the initial stent misdeployment, as fluid instilled into the collection likely also led to colonic expansion, which then mimicked the WON.

A gastrocolostomy is a rare adverse event related to misdeployment of a LAMS intended for a cystogastrostomy 2 . An expanded colon, due to instillation of fluid via a percutaneous drain in the context of a WON-to-colon fistula, may mimic a collection. Maturation of the gastrocolonic fistula prior to LAMS removal avoided the need to manage two sites of perforation, instead facilitating safe and effective dual-sided closure with two OTS clips. Other approaches to management might have included suturing systems or cardiac septal closure devices 3 .

Endoscopy_UCTN_Code_TTT_1AQ_2AG

## Notes

Endoscopy E-Videos https://eref.thieme.de/e-videos
*E-Videos* is an open access online section of the journal *Endoscopy* , reporting on interesting cases and new techniques in gastroenterological endoscopy. All papers include a high-quality video and are published with a Creative Commons CC-BY license. Endoscopy E-Videos qualify for HINARI discounts and waivers and eligibility is automatically checked during the submission process. We grant 100% waivers to articles whose corresponding authors are based in Group A countries and 50% waivers to those who are based in Group B countries as classified by Research4Life (see: https://www.research4life.org/access/eligibility/ ). This section has its own submission website at https://mc.manuscriptcentral.com/e-videos .
